# Lifestyle factors associated with underweight among Japanese adolescents: a cross-sectional study

**DOI:** 10.1186/s13690-017-0213-9

**Published:** 2017-10-23

**Authors:** Hirotaka Ochiai, Takako Shirasawa, Hinako Nanri, Rimei Nishimura, Shohei Nomoto, Hiromi Hoshino, Akatsuki Kokaze

**Affiliations:** 1 Department of Public Health, Showa University School of Medicine, 1-5-8 Hatanodai, Shinagawa-ku, Tokyo, 142-8555 Japan; 2 Division of Diabetes, Metabolism and Endocrinology, Department of Internal Medicine, Jikei University School of Medicine, 3-25-8 Nishi-Shinbashi, Minato-ku, Tokyo, 105-8461 Japan

**Keywords:** Underweight, Lifestyle, Adolescents, A population-based epidemiological study

## Abstract

**Background:**

Because underweight in adolescents poses several health problems, it is important to prevent it in adolescence. For the prevention of underweight, it is necessary to investigate risk factors, especially lifestyles, because these can be modified. However, a limited number of studies exist regarding lifestyle factors associated with underweight among adolescents. The present study aimed to investigate the relationship between lifestyle factors and underweight among Japanese adolescents.

**Methods:**

Study subjects comprised 2641 seventh-grade school children (age 12 or 13 years) from the town of Ina, Saitama Prefecture, Japan. Measurements of height and weight were made for each subject, while information regarding lifestyles of each subject was obtained using a self-administered questionnaire. Underweight was determined by the age- and sex-specific body mass index cut-off points. A logistic regression model was used to estimate the odds ratio (OR) and 95% confidence interval (95% CI) for underweight.

**Results:**

Adolescents who ate slowly were more likely to be underweight than those who did not eat slowly; the OR (95% CI) was 2.78 (1.77–4.39) in boys and 2.69 (1.81–3.98) in girls. Girls who did not exercise were more likely than those who exercised to be underweight (OR: 1.64, 95% CI: 1.07–2.51).

**Conclusions:**

The present study showed that eating slowly and exercise were associated with underweight among Japanese adolescents. These results suggest that eating speed and exercise need to be considered in school health programs for healthy body weight.

## Background

Underweight in children and adolescents poses a considerable public health problem internationally [1]. For example, an underweight status in adolescents was shown to be associated with scoliosis, osteoporosis, pubertal delay, and psychiatric disorders [2]. In addition, it was recently reported that underweight in adolescents is associated with poor perceived health [3]. Thus, underweight in children and adolescents is a serious health condition with substantial consequences for development, health, and well-being [4].

A previous study showed that the tracking correlation for body mass index (BMI) between ages 13 and 19 was 0.85 [5]. This finding suggests that underweight adolescents are likely to be underweight in young adulthood. Therefore, it is important to prevent underweight in adolescents for their health during both adolescence and adulthood, particularly in Japan, where the prevalence of underweight has increased among adolescents during recent years [6, 7].

For prevention of underweight, it is important to investigate risk factors, especially lifestyles, because these can be modified. Lifestyle factors are associated with BMI; for instance, physical activity and eating behaviors were reported to be associated with BMI [8–10]. Therefore, it is necessary to consider both physical activity and eating behaviors when examining lifestyle factors associated with underweight. Although the relationship between physical activity and underweight among adolescents has been investigated [11, 12], eating behaviors such as eating speed, snacking, and breakfast were not considered in these studies. Because previous studies have reported that eating behaviors such as snacking, skipping breakfast, and eating speed were associated with overweight/obesity [13–15], we hypothesized that underweight adolescents were less likely to snack and skip breakfast and were more likely to eat slowly.

Accordingly, the aim of the present study was to investigate the association of lifestyle factors including physical activity and eating behaviors with underweight among Japanese adolescents.

## Methods

### Study subjects

Study subjects comprised 2641 seventh-grade school children (age 12 or 13 years) from all three Ina-town’s junior high schools between 2001 and 2008 (the number of children: 298 in 2001, 317 in 2002, 293 in 2003, 315 in 2004, 312 in 2005, 354 in 2006, 380 in 2007, and 372 in 2008). The town of Ina is in the southern part of Saitama Prefecture, within a 40 km from the center of Tokyo, Japan. As a part of community health services, the town had conducted a questionnaire survey and physical examination for seventh graders in the town’s junior high schools. Details are described elsewhere [16, 17]. This study was conducted based on that survey and examination.

### Questionnaire survey

A self-administered questionnaire was distributed to each subject in junior high school. The questionnaire consisted of two sections: one (on the front of the questionnaire) to be completed by the subject and the other one (on the back) to be filled in by the parent or guardian.

Each subject was asked to complete the questionnaire regarding his or her sex, age, exercise out of physical education class (daily, sometimes, or none), snacking after dinner (always, often, seldom, or none), snacking while watching television or reading books (yes or no), and eating speed (slow, medium, or fast). Subjects were asked to fill in their age. Eating speed was categorized into the following two groups: eating slowly (slow) and not eating slowly (medium or fast).

The questionnaire asked the parent or guardian of each subject about the subject’s birthweight, frequency of eating breakfast (daily, sometimes, or none). Birthweight was dichotomized as <2500 g or ≥2500 g [18]. Frequency of eating breakfast was categorized into two groups: skipping breakfast (sometimes or none) and not skipping breakfast (daily).

### Anthropometric measurements and definition of underweight

In a physical examination, measurements of height and weight of each subject were made either in the school’s infirmary or in a designated room to protect the privacy of subjects. Height was measured to the nearest 0.1 cm using a stadiometer, and body weight was measured to the nearest 0.1 kg using a scale. For anthropometric measurements, subjects wore light clothing and were barefoot. Standardization of the operators for anthropometric measurements was performed. The same measurement protocol was used annually throughout the study period.

BMI was calculated as weight (kg) divided by height (m) squared. Subjects were classified into one of three categories (underweight, normal weight, and overweight/obesity) according to the age- and sex-specific BMI cut-off points that linked to an adult BMI of <18.5, 18.5–24.9, and ≥25.0, proposed by Cole et al. [19, 20]. In addition, the BMI standard deviation score (BMI-SDS) was calculated by the LMS method using the International Obesity Task Force reference values [21].

### Data analysis

The Shapiro-Wilk test was used to test the normality of distribution for each continuous variable. Because most continuous variables were not normally distributed, data are presented as median (25, 75th percentile) for continuous variables or number (%) for categorical variables. Either the Wilcoxon rank-sum or the chi-square test was used to compare various characteristics between two groups (boys vs. girls and underweight group vs. normal weight group). To evaluate the relationship between lifestyle factors and underweight, a logistic regression model was used to estimate the odds ratio (OR) and 95% confidence interval (95% CI) for underweight. In the model, age, birthweight, and all lifestyle variables were included for adjustment because age and birthweight were reported to be associated with BMI [22–24]. For adjustment, age was included in the model as a continuous variable, while the other variables were put in the model as categorical variables. Sex was adjusted for in the analysis of total participants. The test for trend was performed by including “the explanatory variable (exercise) that was coded by ordinal numbers (1 for daily, 2 for sometimes, and 3 for none)” in the model [25]. A *P* value <0.05 was considered statistically significant. All statistical analyses were performed using Statistical Analysis System (SAS) software (version 9.4; SAS Institute Inc., Cary, NC, USA).

## Results

Among all 2641 subjects, 28 refused to participate in the questionnaire survey and physical examination (participation rate: 98.9%), and 263 were excluded due to missing data about variables in the present study. Thus, data from 2350 subjects were analyzed.

Table 1 shows characteristics of study participants by sex. Boys were significantly higher and heavier than girls. BMI-SDS was significantly higher in boys than in girls. The prevalence of overweight/obesity was higher in boys than in girls, while the prevalence of underweight was higher in girls than in boys. There was a statistically significant association between boys and girls in exercise. A statistically significant association between boys and girls was found in the proportion of those who answered “Yes” to the questions about skipping breakfast or snacking while watching television or reading books. A significantly higher proportion of girls reported eating slowly compared with boys.

**Table 1:** Characteristics of study participants by sex (Japan, 2001–2008)

	Boys (*N* = 1211)	Girls (*N* = 1139)	*P* value^a^
Age (years)	12.0 (12.0, 13.0)	12.0 (12.0, 13.0)	0.822
Bitrhweight (g), *n* (%)			
< 2500	77 (6.4)	85 (7.5)	0.291
≥ 2500	1134 (93.6)	1054 (92.5)	
Height (cm)	155.6 (149.2, 161.0)	153.3 (149.4, 157.2)	<0.001
Weight (kg)	44.3 (38.8, 50.8)	43.5 (38.9, 48.1)	0.007
BMI (kg/m^2^)	18.2 (16.8, 20.0)	18.3 (16.9, 20.0)	0.389
BMI-SDS	0.25(−0.35, 0.89)	0.15 (−0.44, 0.75)	0.002
Physique, *n* (%)			
Underweight	99 (8.2)	127 (11.2)	0.005
Normal weight	933 (77.0)	882 (77.4)	
Overweight/obesity	179 (14.8)	130 (11.4)	
Exercise, *n* (%)			
Daily	1003 (82.8)	668 (58.7)	<0.001
Sometimes	94 (7.8)	157 (13.8)	
None	114 (9.4)	314 (27.6)	
Skipping breakfast, *n* (%)			
Yes	58 (4.8)	84 (7.4)	0.009
No	1153 (95.2)	1055 (92.6)	
Snacking after dinner, *n* (%)			
Always or often	735 (60.7)	659 (57.9)	0.162
Seldom or none	476 (39.3)	480 (42.1)	0.015
Snacking while watching television or reading books, *n* (%)			
Yes	482 (39.8)	510 (44.8)	0.015
No	729 (60.2)	629 (55.2)	
Eating slowly, *n* (%)			
Yes	207 (17.1)	279 (24.5)	<0.001
No	1004 (82.9)	860 (75.5)	

*BMI* body mass index, *BMI-SDS* body mass index standard deviation score

Except where indicated *n* (%), values are expressed as median (25, 75th percentile)

^a^Wilcoxon rank-sum test or chi-square test

Comparisons of characteristics between the normal weight group and the underweight group in boys are shown in Table 2. Anthropometric variables were significantly lower in the underweight group than in the normal weight group. The proportion of those who ate slowly in the underweight group was significantly higher than that in the normal weight group.

**Table 2:** Comparisons of characteristics between the normal weight and underweight groups in boys (Japan, 2001–2008)

	Normal weight (*n* = 933)	Underweight (*n* = 99)	*P* value^a^
Age (years)	12.0 (12.0, 13.0)	12.0 (12.0, 13.0)	0.186
Birthweight (g), *n* (%)			
< 2500	50 (5.4)	10 (10.1)	0.055
≥ 2500	883 (94.6)	89 (89.9)	
Height (cm)	155.5 (149.2, 160.8)	149.9 (144.6, 154.7)	<0.001
Weight (kg)	43.7 (39.1, 48.3)	33.0 (30.8, 36.2)	<0.001
Body mass index (kg/m^2^)	18.0 (16.9, 19.2)	15.0 (14.6, 15.3)	<0.001
Exercise, *n* (%)			
Daily	788 (84.5)	81 (81.8)	0.325
Sometimes	71 (7.6)	6 (6.1)	
None	74 (7.9)	12 (12.1)	
Skipping breakfast, *n* (%)			
Yes	45 (4.8)	3 (3.0)	0.421
No	888 (95.2)	96 (97.0)	
Snacking after dinner, *n* (%)			
Always or often	580 (62.2)	57 (57.6)	0.372
Seldom or none	353 (37.8)	42 (42.4)	
Snacking while watching television or reading books, *n* (%)			
Yes	383 (41.1)	34 (34.3)	0.196
No	550 (59.0)	65 (65.7)	
Eating slowly, *n* (%)			
Yes	161 (17.3)	35 (35.4)	<0.001
No	772 (82.7)	64 (64.7)	

Except where indicated *n* (%), values are expressed as median (25, 75th percentile)

^a^Wilcoxon rank-sum test or chi-square test

Table 3 shows comparisons of characteristics between the normal weight group and the underweight group in girls. Anthropometric variables in the underweight group were significantly lower than those in the normal weight group. There was a statistically significant association between the normal weight and the underweight group in exercise. The proportion of those who ate slowly was significantly higher in the underweight group compared with the normal weight group.

**Table 3:** Comparisons of characteristics between the normal weight and underweight groups in girls (Japan, 2001–2008)

	Normal weight (*n* = 882)	Underweight (*n* = 127)	*P* value^a^
Age (years)	12.0 (12.0, 13.0)	12.0 (12.0, 13.0)	0.017
Birthweight (g), *n* (%)			
< 2500	77 (8.7)	6 (4.7)	0.125
≥ 2500	805 (91.3)	121 (95.3)	
Height (cm)	153.4 (149.6, 157.1)	151.3 (147.1, 155.5)	<0.001
Weight (kg)	43.5 (39.7, 46.9)	34.7 (32.3, 37.2)	<0.001
Body mass index (kg/m^2^)	18.3 (17.2, 19.5)	15.3 (14.7, 15.5)	<0.001
Exercise, *n* (%)			
Daily	547 (62.0)	65 (51.2)	0.033
Sometimes	115 (13.0)	17 (13.4)	
None	220 (24.9)	45 (35.4)	
Skipping breakfast, *n* (%)			
Yes	63 (7.1)	7 (5.5)	0.499
No	819 (92.9)	120 (94.5)	
Snacking after dinner, *n* (%)			
Always or often	509 (57.7)	76 (59.8)	0.649
Seldom or none	373 (42.3)	51 (40.2)	
Snacking while watching television or reading books, *n* (%)			
Yes	397 (45.0)	58 (45.7)	0.889
No	485 (55.0)	69 (54.3)	
Eating slowly, *n* (%)			
Yes	203 (23.0)	56 (44.1)	<0.001
No	679 (77.0)	71 (55.9)	

Except where indicated *n* (%), values are expressed as median (25, 75th percentile)

^a^Wilcoxon rank-sum test or chi-square test

Adjusted ORs of lifestyle factors for underweight and their 95% CIs were calculated in each sex (Table 4 in boys and Table 5 in girls). Boys who ate slowly were more likely to be underweight than those who did not eat slowly (OR: 2.78, 95% CI: 1.77–4.39). Girls who did not exercise were more likely than those who exercised to be underweight (OR: 1.64, 95% CI: 1.07–2.51), and there was a significant dose-response relationship between the frequency of exercise and underweight (*P* for trend = 0.021). Girls who ate slowly were more likely to be underweight than those who did not eat slowly (OR: 2.69, 95% CI: 1.81–3.98).

**Table 4:** Adjusted odds ratios and 95% confidence intervals for underweight in boys (Japan, 2001–2008)

Lifestyle variables	Total *N*	Underweight *n* (%)	AOR (95% CI)	*P* value
Exercise				
Daily	869	81 (9.3)	1.00	
Sometimes	77	6 (7.8)	0.89 (0.37–2.15)	0.802
None	86	12 (14.0)	1.73 (0.88–3.38) *P* for trend = 0.173	0.111
Skipping breakfast				
Yes	48	3 (6.3)	0.70 (0.21–2.36)	0.568
No	984	96 (9.8)	1.00	
Snacking after dinner				
Always or often	637	57 (9.0)	0.89 (0.57–1.38)	0.593
Seldom or none	395	42 (10.6)	1.00	
Snacking while watching television or reading books				
Yes	417	34 (8.2)	0.71 (0.45–1.12)	0.143
No	615	65 (10.6)	1.00	
Eating slowly				
Yes	196	35 (17.9)	2.78 (1.77–4.39)	<0.001
No	836	64 (7.7)	1.00	

*AOR* adjusted odds ratio, *CI* confidence interval

Age (years), birthweight (<2500 g or ≥2500 g), and all lifestyle variables were included for adjustment

**Table 5:** Adjusted odds ratios and 95% confidence intervals for underweight in girls (Japan, 2001–2008)

Lifestyle variables	Total *N*	Underweight *n* (%)	AOR (95% CI)	*P* value
Exercise				
Daily	612	65 (10.6)	1.00	
Sometimes	132	17 (12.9)	1.34 (0.75–2.39)	0.331
None	265	45 (17.0)	1.64 (1.07–2.51) *P* for trend = 0.021	0.022
Skipping breakfast				
Yes	70	7 (10.0)	0.67 (0.29–1.55)	0.352
No	939	120 (12.8)	1.00	
Snacking after dinner				
Always or often	585	76 (13.0)	1.08 (0.73–1.60)	0.714
Seldom or none	424	51 (12.0)	1.00	
Snacking while watching television or reading books				
Yes	455	58 (12.8)	0.97 (0.65–1.43)	0.867
No	554	69 (12.5)	1.00	
Eating slowly				
Yes	259	56 (21.6)	2.69 (1.81–3.98)	<0.001
No	750	71 (9.5)	1.00	

*AOR* adjusted odds ratio, *CI* confidence interval

Age (years), birthweight (<2500 g or ≥2500 g), and all lifestyle variables were included for adjustment

Table 6 showed the adjusted ORs of lifestyle factors for underweight and their 95% CIs in total participants. Adolescents who did not exercise were more likely than those who exercised to be underweight (OR: 1.59, 95% CI: 1.11–2.27), and there was a significant dose-response relationship between the frequency of exercise and underweight (*P* for trend = 0.012). Adolescents who ate slowly were more likely to be underweight than those who did not eat slowly (OR: 2.67, 95% CI: 1.99–3.59). Girls were not more likely than boys to be underweight (OR: 1.16, 95% CI: 0.86–1.56). There were no effect modifications of sex and lifestyle factors on underweight.

**Table 6:** Adjusted odds ratios and 95% confidence intervals for underweight in total participants (Japan, 2001–2008)

Lifestyle variables	Total *N*	Underweight *n* (%)	AOR (95% CI)	*P* value
Exercise				
Daily	1481	146 (9.9)	1.00	
Sometimes	209	23 (11.0)	1.16 (0.72–1.87)	0.546
None	351	57 (16.2)	1.59 (1.11–2.27) *P* for trend = 0.012	0.011
Skipping breakfast				
Yes	118	10 (8.5)	0.69 (0.35–1.37)	0.295
No	1923	216 (11.2)	1.00	
Snacking after dinner				
Always or often	1222	133 (10.9)	0.98 (0.73–1.32)	0.903
Seldom or none	819	93 (11.4)	1.00	
Snacking while watching television or reading books				
Yes	872	92 (10.6)	0.86 (0.64–1.16)	0.322
No	1169	134 (11.5)	1.00	
Eating slowly				
Yes	455	91 (20.0)	2.67 (1.99–3.59)	<0.001
No	1586	135 (8.5)	1.00	

*AOR* adjusted odds ratio, *CI* confidence interval

Sex, age (years), birthweight (<2500 g or ≥2500 g), and lifestyle variables were included in the adjustment

## Discussion

The present study investigated the relationship between lifestyle factors and underweight among Japanese adolescents. In this study, eating slowly and exercise were associated with underweight. To the best of our knowledge, this is the first study regarding the association of lifestyle factors (eating speed and exercise) with underweight among adolescents in Japan. However, the causality between lifestyle factors and underweight should be carefully interpreted because this was a cross-sectional study.

In this study, there were statistically significant differences between boys and girls in some anthropometric variables and lifestyles. A previous study reported that adolescence is characterized by a global acceleration of growth and maturation, with differential changes between both sexes [26]. In addition, a recent study showed that there were statistically significant differences between boys and girls in lifestyle factors such as breakfast, eating speed, and physical activity [14]. Therefore, we analyzed the data separately for each sex to examine the association between lifestyle factors and underweight among adolescents.

In our study, the prevalence of underweight was 8.2% in boys and 11.2% in girls. According to national statistics from Ministry of Education, Culture, Sports, Science and Technology, the prevalence of underweight (defined by percentage of overweight, which is the modified weight-for-height method [27]) in 2008 was 2.3% among boys (ages 12) and 3.9% among girls (ages 12) in Japan [28]. When the underweight in our study was defined by the same criteria using percentage of overweight, the prevalence of underweight in 2008 was 2.6% in boys aged 12 years and 3.4% in girls aged 12 years, which were similar to the results of the national statistics in Japan. A recent study reported that the prevalence of underweight among Dutch boys and girls (ages 12–18) in 2009 was 9.1% and 10.4%, respectively [4]. Elinder et al. reported that the prevalence in a Swedish cohort of boys and girls (mean age: 15.6) in 2002 was 3.5% and 11.1%, respectively [11]. A previous study among German adolescents reported that the prevalence was 12.6% among boys and 19.1% among girls in 2001/2002 [29]. Because the definition of underweight, the year of the data collection, and the age of study subjects differed by studies, future studies will be needed to compare the prevalence of underweight between Japan and other countries.

Eating slowly was significantly associated with underweight in this study. One of the reasons could be due to total energy intake. Murakami et al. showed that rate of eating was positively associated with energy intake, BMI, and the risk of overweight [30]. Otsuka et al. found a positive association between “the rate of eating” and “energy intake and current BMI”, suggesting that eating fast would lead to obesity [31]. Therefore, we hypothesized that total energy intake in the eating slowly group was lower than that in the not eating slowly group, which could lead to lower BMI in the eating slowly group compared with the not eating slowly group in the present study. In fact, BMI among the eating slowly group was lower than that among the not eating slowly group in our study (data not shown). Another possible explanation might be due to appetite. A previous study reported that higher BMI SD scores were associated with lower satiety responsiveness and higher food cue responsiveness [32]. In addition, Webber et al. showed that there was a significant negative association between “satiety responsiveness/slowness in eating” and “physique (underweight, lower healthy weight, higher healthy weight, overweight, and obese)”; the score of satiety responsiveness/slowness in eating among underweight children was higher than that among healthy weight children [33]. These studies [32, 33] suggested that underweight adolescents could have a poor appetite, which leads to eating slowly. However, it is difficult to determine the temporal sequence of eating slowly and underweight in our study because the present study was cross-sectional. Therefore, further studies will be needed to evaluate the causal relationship between eating slowly and underweight.

In the present study, girls who did not exercise were more likely to be underweight than those who exercised. These results persisted even after adjustment for age, birthweight, and all lifestyle variables. Recent studies have shown that low physical activity was associated with underweight [11, 12], which is consistent with the results in this study. One of the reasons for the association between exercise and underweight could be due to the influence of exercise on lean body mass. Elinder et al. showed that physical activity is a major determinant of skeletal muscle growth leading to an increase in lean body mass [11]. In addition, a previous study reported that exercise exerts a positive effect on bone mass [34]. In fact, it was recently shown that total body lean mass among an inactive group was lower than that among average or active groups [35]. Another reason is due to health problems related to underweight. A recent study reported that an underweight status in adolescents was associated with several health problems [2]. These health problems in underweight adolescents could lead to physical inactivity.

There are some limitations to this study. First, information regarding lifestyle factors such as eating speed and exercise was collected by a self-administered questionnaire, which might be not objective. However, a previous study showed a high level of concordance between self-reported and friend-reported rate of eating [10]. In addition, Petty et al. indicated that self-reported eating rate aligned with laboratory-measured eating rate [36]. Moreover, Chen et al. reported that schoolchildren’s self-reported physical activity is in accordance with objective data [37]. Second, the present study results could be affected by some potential confounders. For example, socioeconomic factors were reported to be associated with underweight [29, 38]. Moreover, the social pressure to be thin, body image perception, and gestational age could affect our study findings. Because this information was not considered in the present study, residual confounding cannot be ruled out. However, a recent study showed that Japan is still one of the most egalitarian nations in the world, and social inequalities within the population are less expressed [39]. Therefore, the influence of socioeconomic factors on this study might not be substantial. Third, the age of each subject was indicated as “12 or 13” (years) in this study. Therefore, the age- and sex-specific BMI cut-off points for adolescents aged 12 or 13 years [19, 20] were applied, whereas the cut-off points for those aged 12.5 years were not applied. Finally, our study subjects (the number of children: 298 in 2001, 317 in 2002, 293 in 2003, 315 in 2004, 312 in 2005, 354 in 2006, 380 in 2007, and 372 in 2008) were from one town, which might limit generalizability to other populations.

## Conclusions

The present study showed that eating slowly and exercise were associated with underweight among Japanese adolescents. The results suggest that eating speed and exercise need to be considered in school health programs for healthy body weight, although future studies are needed to verify our findings.

## Abbreviations

BMI: Body mass index
CI: Confidence interval
OR: Odds ratio
